# A biologically inspired spiking neural P system in selective visual attention for efficient feature extraction from human motion

**DOI:** 10.3389/frobt.2022.1028271

**Published:** 2022-09-23

**Authors:** Esteban Anides, Luis Garcia, Giovanny Sanchez, Juan-Gerardo Avalos, Marco Abarca, Thania Frias, Eduardo Vazquez, Emmanuel Juarez, Carlos Trejo, Derlis Hernandez

**Affiliations:** ^1^ Instituto Politecnico Nacional ESIME Culhuacan, Mexico City, Mexico; ^2^ Tecnologico Nacional de Mexico, Tecnologico de Estudios Superiores de Ecatepec, Estado de Mexico, Mexico

**Keywords:** spiking neural P systems, selective visual attention, human action detection, human visual perception, communication on request, astrocyte-like control, rules on the synapses

## Abstract

Nowadays, human action recognition has become an essential task in health care and other fields. During the last decade, several authors have developed algorithms for human activity detection and recognition by exploiting at the maximum the high-performance computing devices to improve the quality and efficiency of their results. However, in real-time and practical human action recognition applications, the simulation of these algorithms exceed the capacity of current computer systems by considering several factors, such as camera movement, complex scene and occlusion. One potential solution to decrease the computational complexity in the human action detection and recognition can be found in the nature of the human visual perception. Specifically, this process is called selective visual attention. Inspired by this neural phenomena, we propose for the first time a spiking neural P system for efficient feature extraction from human motion. Specifically, we propose this neural structure to carry out a pre-processing stage since many studies have revealed that an analysis of visual information of the human brain proceeds in a sequence of operations, in which each one is applied to a specific location or locations. In this way, this specialized processing have allowed to focus the recognition of the objects in a simpler manner. To create a compact and high speed spiking neural P system, we use their cutting-edge variants, such as rules on the synapses, communication on request and astrocyte-like control. Our results have demonstrated that the use of the proposed neural P system increases significantly the performance of low-computational complexity neural classifiers up to more 97% in the human action recognition.

## Introduction

Nowadays, human action recognition (HAR) has become a vital task in many applications, such as intelligent human–machine interfaces, intelligent video surveillance, video storage and retrieval and home monitoring (Liu J. et al., 2019). Specifically, remote patient monitoring (RPM) has emerged as a potential solution for rehabilitation purposes since the number of patients with stroke or other disability has increased significantly. Therefore, human action recognition plays an important role in this application since the movement of patients needs to be continuously monitored and rectified. In this way, their motion patterns can be corrected. Nowadays, current IoT technologies, such as RPM, glucose monitoring, heart-rate monitoring, depression and mood monitoring, among others, have allowed to the doctors take care of the health of their patients from home (Liu Z. et al., 2019; Bouchabou et al., 2021). In particular, RPM technology have allowed to therapists monitor the patients, who are in a home-based rehabilitation scheme, remotely by using broadband internet (Hasan et al., 2019; Farias et al., 2020). Until date, many algorithms have developed to detect and recognize the human motion under controlled environments to achieve high accuracy. Despite this, the simulation of these advanced algorithms overcomes the computational capacity of the current computing systems. In addition, bandwidth congestion may ocurr when trying to send multiple videos to the doctor. As a consequence, the detection and recognize of the human motion in real-time becomes infeasible since the latency of the RPM system is increased significantly. In the past decades, several approaches have been developed to efficiently perform human action recognition (Baccouche et al., 2011; Ji et al., 2012; Grushin et al., 2013; Ali and Wang, 2014; Liu et al., 2014; Shu et al., 2014; Shi et al., 2015; Veeriah et al., 2015; Dash et al., 2021). In general terms, these approaches intend to determine when the action is occurring and what is this action by considering the starting and ending times of all action occurrences from the video. Despite achieving these important developments, there are still several challenges to overcome. In particular, most of these approaches show high performance in terms of recognition accuracy at the cost of exhibiting high computational complexity. As a consequence, the use of these approaches in practical real-time RPM application can be limited. Therefore, there is still the need to develop low-computational complexity algorithms with high recognition accuracy. One potential solution can be found in the nature. In particular, in the human vision in which a compact representation of the visual content in the form of salient objects/events can be obtained. Recently, advanced approaches have been developed based on the visual attention. For example, (Sharma et al., 2015) presented a visual attention algorithm based on multi-layered recurrent neural networks (RNNs) to efficiently perform action recognition in videos. However, the model exhibits high computational complexity by performing a large number of convolutional operations. Other example can be found in Guo et al. (2022). The authors developed a linear attention method named large kernel attention (LKA) for visual tasks. In general terms, these works intend to improve the recognition accuracy by increasing their computational complexity. From the engineering perspective, this factor is vital since most of the current processing devices are portable. Therefore, the simulation of these approaches exceeds the computational capabilities of these devices. Here, we propose for the first time a method to create an efficient pre-processing step inspired by the visual attention mechanism. In this way, any classification scheme can be used to improve their performance significantly. In this way, the human action recognition can be performed efficiently by ensuring a low computational complexity.

## Materials and methods

### The proposed pre-processing visual system based on spiking neural P systems

**FIGURE 1:**
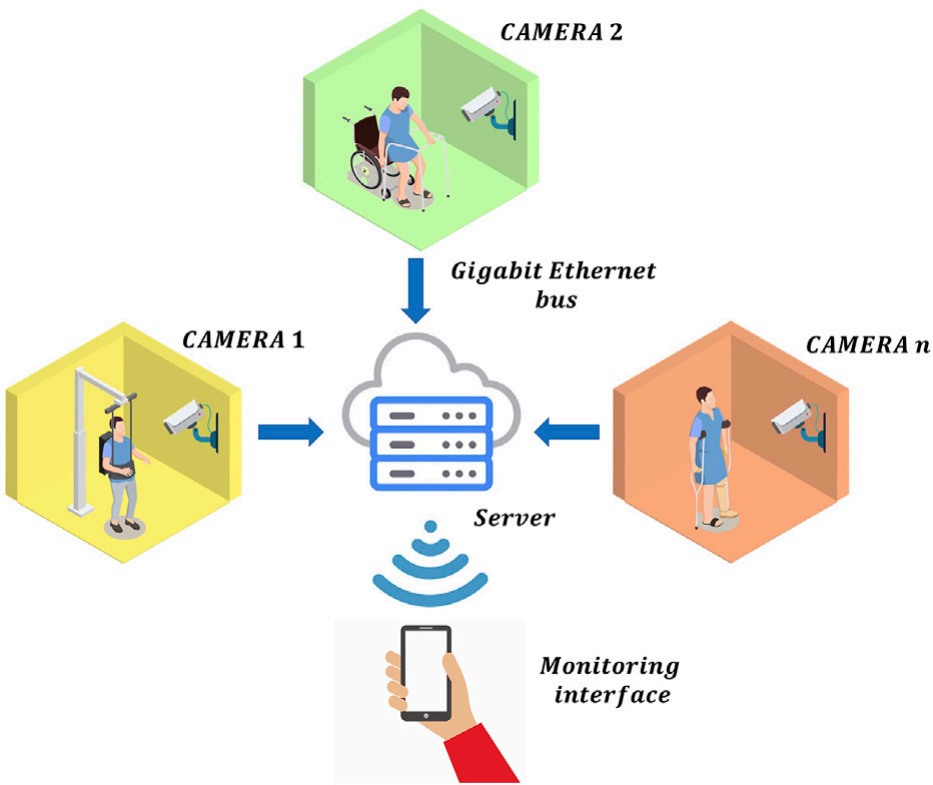
A conventional remote patient monitoring scheme.

**FIGURE 2:**
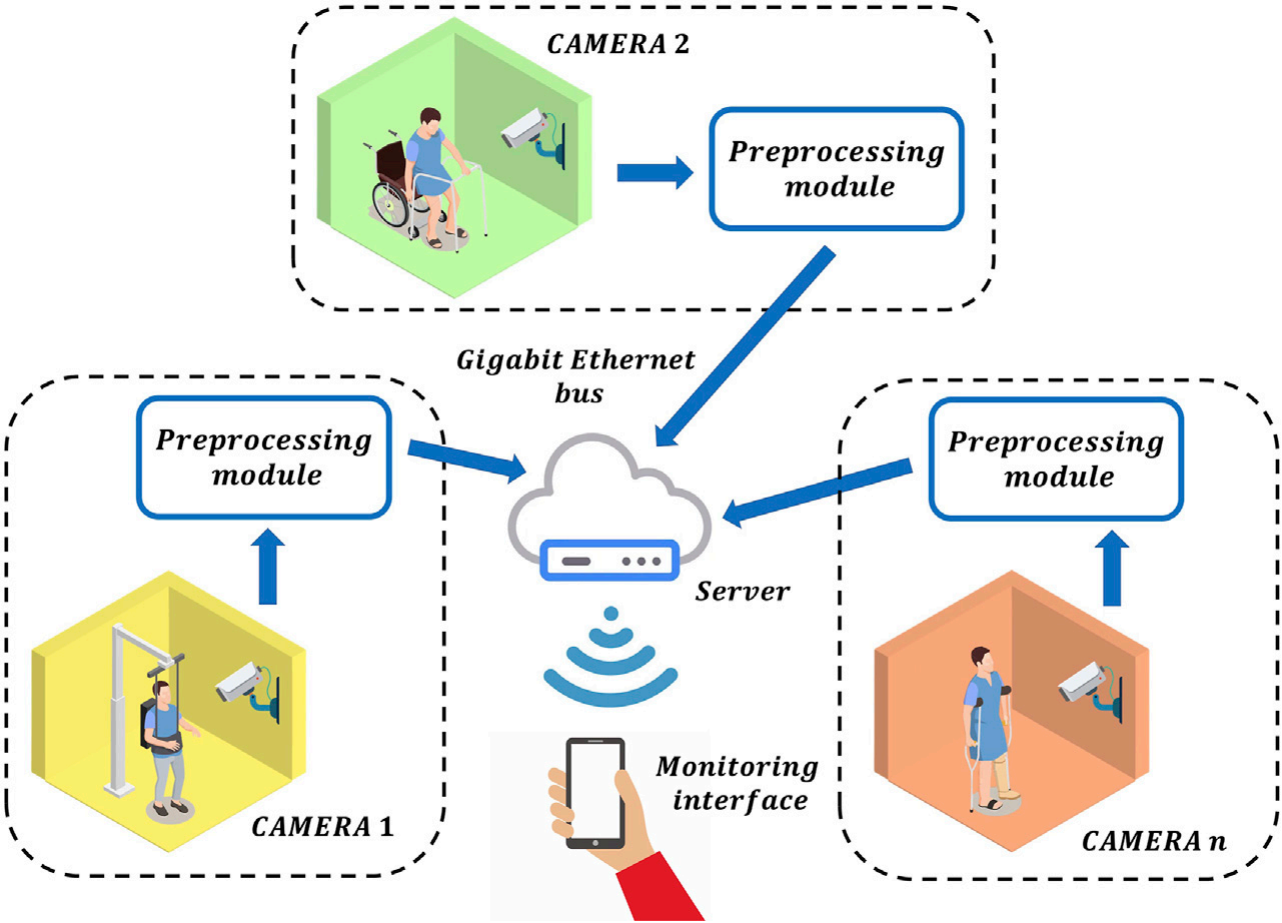
The proposed remote patient monitoring scheme using local pre-processing visual system based on spiking neural P systems.

Nowadays, several patients can be monitored remotely by using the current IoT technologies, as shown in Figure 1. Under this RPM scheme, the information of each patient is sent to the doctor via Gigabit Ethernet. In general, this information is composed of frames of the whole scene with ultra-high definition, which implies to send billions of bits at each second per each patient to the server. Therefore, a bandwidth congestion can ocurr by monitoring multiple patients. Obviously, this information can be compressed by using different video data compression methods. However, the latency of the RPM scheme is increased significantly by performing this additional process. Therefore, the monitoring of the patients in real-time is unfeasible. To avoid this, one potential solution can be found if each local monitoring system only sends the relevant information to detect the movement of the patients instead of sending the whole scene. Inspired by the selective attention, we propose a model of pre-processing before data analysis and learning. This neural behaviour can be found in the human visual system since this obtains a compact representation of the visual content in the form of salient events/objects. In general, when the visual system detects a change in position or pose in an observed object, it produces a different pattern of activity from a population of neurons, corresponding to a different visual association response (DiCarlo et al. (2012)). By exploiting these advantages to the maximum, we propose a RPM scheme using local pre-processing visual system based on spiking neural P (SN P) systems, as shown in Figure 2. In this system, the pre-processing stage can be performed by an embedded system, which only sends the relevant information to the doctor in real-time. The SN P systems were proposed as a new class of distributed and parallel systems (Ionescu et al. (2006)). Nowadays, some authors have proposed novel variants of SN P systems such as weighted synapses (Pan et al. (2012)), with communication on request (Pan et al. (2017)) and rules on synapses (Song et al. (2014)). These systems have had a great impact, since they have proven to be useful in engineering applications.

Before presenting the proposed remote patient monitoring scheme, we provide the formal definition of SN P systems (Π) of degree *m* ≥ 1, as follows:

Π = {*O*, *σ*_1_, *σ*_2_, …, *σ*_*m*_, *syn*, *in*, *out*}

Where

1) *O* = {*a*} is the singleton alphabet, where *a* is called *spike*;
2) *σ*_1_, *σ*_2_, …, *σ*_*m*_ are neurons of the form *σ*_*i*_ = (*n*_*i*_, *R*_*i*_)

     where

a) *n*_*i*_ ≥ 0 is the initial number of spikes contained in neuron *σ*_*i*_;
b) *R*_*i*_ is the finite set of rules of the following two forms:
(1) *E*/*a* ^*c*^ → *a*, where *E* is a regular expression over alphabet *O*, *c* ≥ 1;
(2) *a* ^*s*^ → *λ*, for *s* ≥ 1 and *a* ^*s*^∉*L*(*E*), where *λ* represents the empty string;
3) *syn* is a set of synapses, where each element is given by:

(*i*, *j*) ⊆{1, …, *m*}×{1, …, *m*},Where

a) *m* is the total number of neurons (*σ*);
b) *i*, *j* denote pre-synaptic and post-synaptic neurons, respectively (1 ≤ *i*, *j* ≤ *m*).

Here, synapses are defined as follows:

$$syn=\left(\sigma_{i},\sigma_{j},\Delta t_{d},R\right)$$

a) Δ*t*_*d*_ defines the maximum number of steps required for a spike to reach neuron *j* via neuron *i* (Garcia et al. (2021)).
b) *R*_(*i*,*j*)_ is a finite set of rules of the following two forms:
• *E*/*a* ^*c*^ → *a* ^*p*^; *d* (*firing rule*), where *E* is a regular expression over *O*, *c* ≥ *p* ≥ 1 and *d* ≥ 0.
• *a* ^*s*^ → *λ* (*forgetting rule*), for some *s* ≥ 1, with the restriction that *a* ^*s*^∉*L*(*E*) for any rule *E*/*a* ^*c*^ → *a* ^*p*^; *d* from any *R*_(*i*,*j*)_.
4) *in*, *out* ∈ {1, …, *m*} indicates the input and the output neurons, respectively.

**FIGURE 3:**
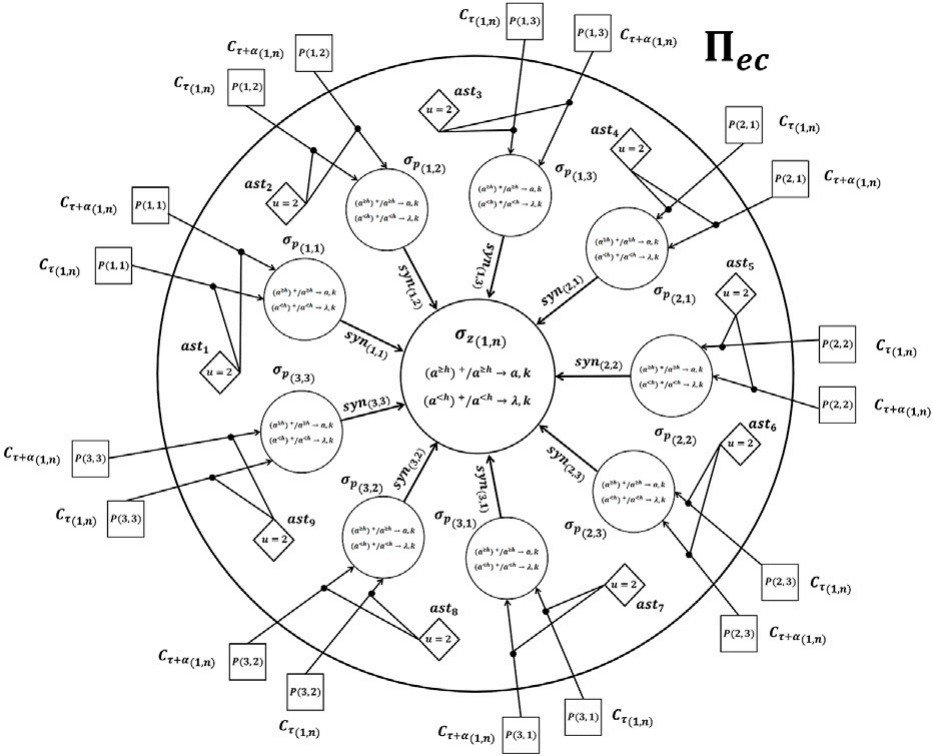
The general scheme of a basic processing unit, Π_*ec*_, with hierarchical structure.

Once the formal definition was provided, we present the structure of the spiking neural P system, as shown in Figure 3. Here, we take inspiration of the neural activity at all levels of the visual system from the retina to regions of parietal and frontal cortex. Specifically, neurons in early visual areas have small spatial receptive fields (RFs), and neurons in later areas have large RFs and code abstract features such as behavioral relevance. In this way, the information is organized to obtain local and global features of a image. Therefore, selective attention coordinates the activity of neurons to compact representations of an object. Inspired by this neural behaviour, we build a SN P system with two layers. The first layer, which is composed of neurons, *σ*_*p*_, intends to mimic the behaviour of the small spatial receptive fields, and the second layer is composed of a set of neurons, $\sigma_{z_{\left(m,n\right)}}$ .

Based on the hierarchical structure of the proposed processing unit, Π_*ec*_, the definition can be expressed as follows:

$$\begin{array}{ll} \Pi_{ec} & =\left\{0,\sigma_{z},\sigma_{p_{\left(1,1\right)}},\ldots ,\sigma_{p_{\left(3,3\right)}},ast_{1},\ldots ,ast_{9},in,out\right\}\\ 0 & =\left\{a\right\}\\ \sigma_{p_{i,j}}|\left(i,j\right)\in \left\{\left(1,1\right),\left(1,2\right),\ldots ,\left(n,m\right)\right\} & =\left(0,R_{p_{1}},R_{p_{2}}\right)\\ \sigma_{z} & =\left(0,R_{z_{1}},R_{z_{2}}\right)\\ R_{p_{1}} & =\left\{{\left(a^{\geq h}\right)}^{+}/a^{\geq h}\to a,k\right\},\\ R_{p_{2}} & =\left\{{\left(a^{<h}\right)}^{+}/a^{<h}\to \lambda ,k\right\}\\ R_{z_{1}} & =\left\{{\left(a^{\geq h^{\prime }}\right)}^{+}/a^{\geq h^{\prime }}\to a,k\right\},\\ R_{z_{2}} & =\left\{{\left(a^{<h^{\prime }}\right)}^{+}/a^{<h^{\prime }}\to \lambda ,k\right\}\\ in & =\left\{P_{\left(1,1\right)},P_{\left(1,2\right)},\ldots ,P_{\left(n,m\right)}\right\}\\ out & =\left\{\sigma_{z}\right\}\\ ast_{i}|i\in \left\{0,1,\ldots ,9\right\} & =\left\{u=2,syn_{1_{i}},syn_{2_{i}}\right\} \end{array}$$

**FIGURE 4:**
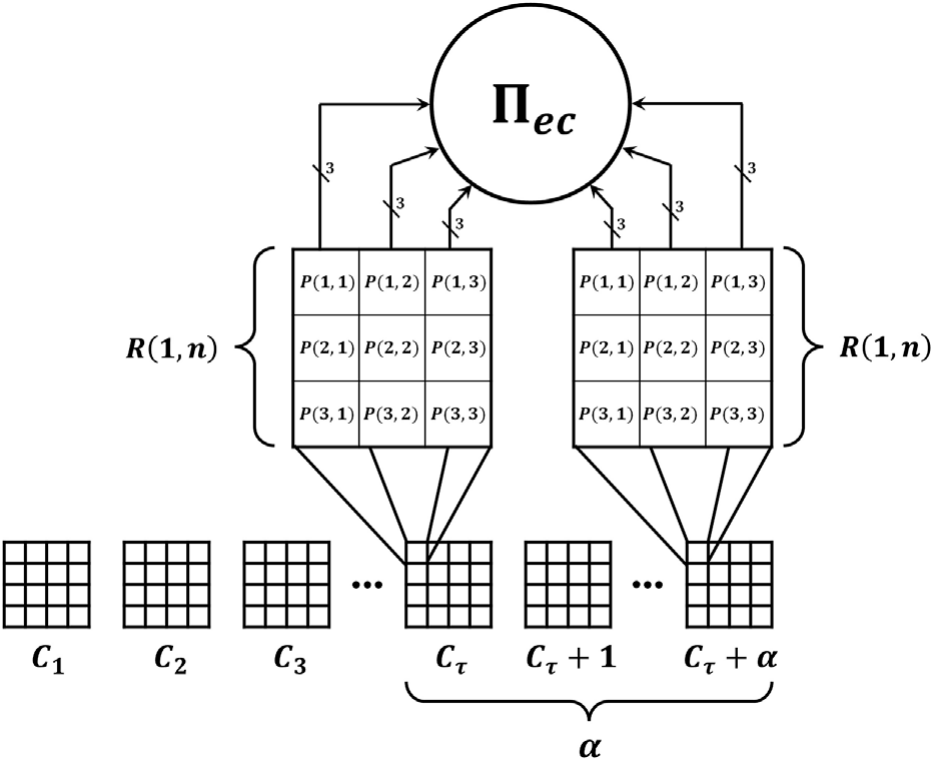
The proposed video processing system using multiple basic processing units, Π_*ec*_ in parallel.

The proposed processing unit, Π_*ec*_, with hierarchical structure works as follows:

Initially, the user must define the distance between several frames by means of the factor, *α*, and the number of regions, *R*, per each frame, *C*, as shown in Figure 4. Specifically, the distance, *α*, between frames, *C*, defines the speed at which the video is analyzed. On the other hand, the regions define the area of analysis within each frame, *C*. Therefore, each region of a frame, *C*, represents a part of the human body. Once the parameters, *α*, and *R* were defined, the value of each pixel is converted into its equivalent number of spikes. For example, if the pixel value is equal to 5, this value is represented by means of a train of five spikes. Once the pixels are converted into spikes, these are sent to the first layer of neurons, *σ*_*p*_. As can be seen in Figure 3, neurons, *σ*_*p*_, have astrocytes, *ast*, to regulate the input spikes. Here, these astrocytes performs an intrinsic subtraction by means of their inhibition rule. Otherwise, only one spike is sent to its respective neuron, *σ*_*p*_. The use of this mechanism has allowed us to perform the comparison between two frames, *C*. In this way, the movement of the human can be detected, i.e., if there is a difference between two consecutive frames, there is significant movement of the human. Therefore, only significant information can be processed by neurons, *σ*_*p*_ and the remaining information is discard. Once the input pulses are delivered to neurons, *σ*_*p*(*i*,*j*)_, these neurons fires the accumulated spikes to neuron, *σ*_*z*_. In general, neurons, *σ*_*z*_ were proposed to indicate the motion of a particular region, *R*, by applying their firing rules, $\left({\left(a^{\geq h}\right)}^{+}/a^{\geq h}\to a,k\right)$ , where *k* is in function of the bit-resolution of the image, and *h* is a threshold, which is defined in terms of the number of pixels per each region, *R*. Therefore, this threshold is used as descriptor to indicate the information of the spatial location of regions, *R*, with significant movement.

## Results

**FIGURE 5:**
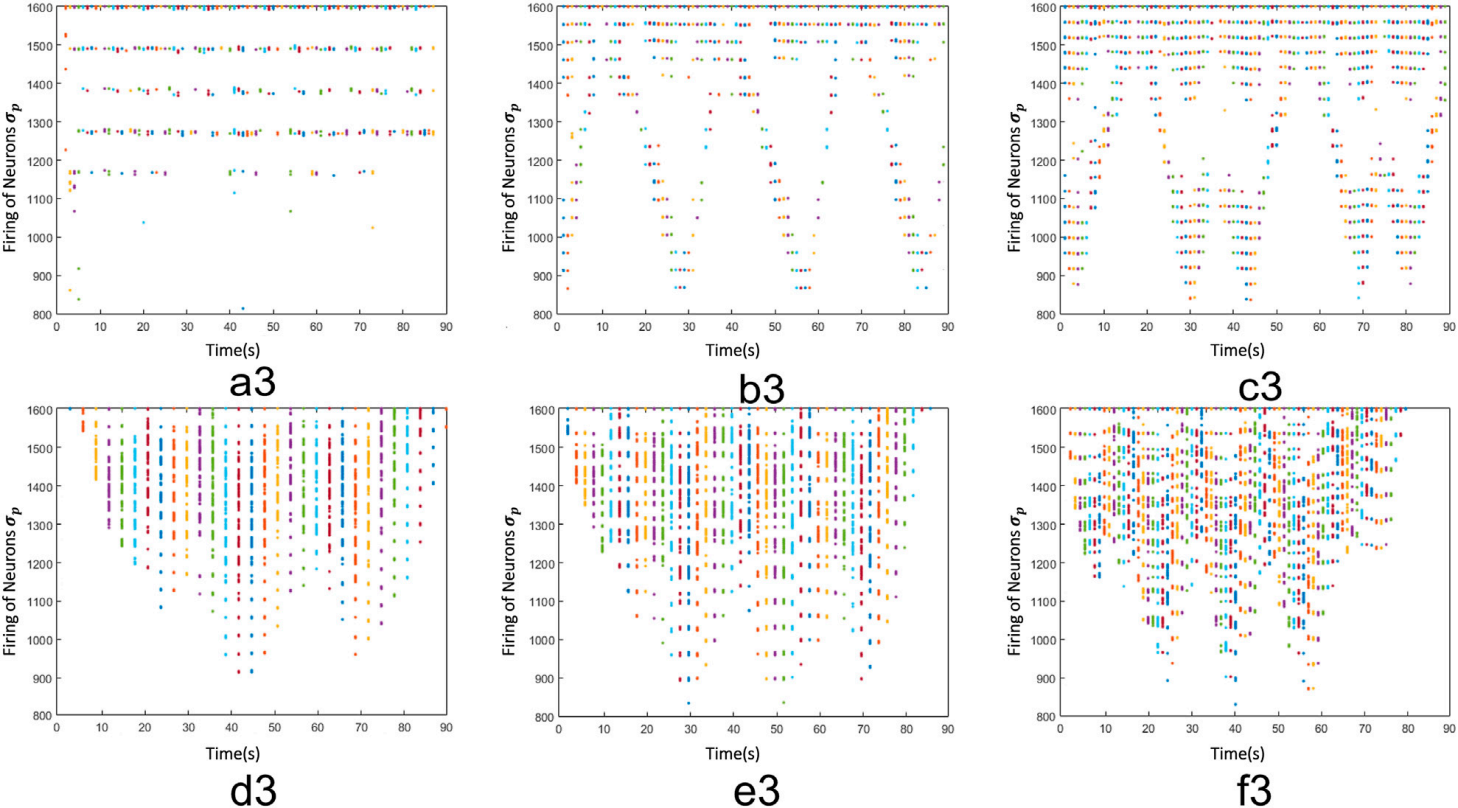
Firing patterns of actions from the KTH dataset, where **a3, b3, c3, d3, e3 and f3** show the firing patterns of the neurons *σ*_*p*_, which represent the motions boxing, hand clapping, hand waving, jogging, running and walking respectively.

To demonstrate the performance of the proposed processing units, Π_*ec*_, we use the KTH dataset (Schuldt et al., 2004). In this way, we make a coherent comparison between our approach and existing works (Baccouche et al., 2011; Ji et al., 2012; Grushin et al., 2013; Ali and Wang, 2014; Liu et al., 2014; Shu et al., 2014; Shi et al., 2015; Veeriah et al., 2015; Dash et al., 2021). This data set is composed of 6 videos, which involves human actions, such as boxing(a), hand clapping(b), hand waving(c), jogging(e), running (d) and walking(f). These activities were carried out by 25 people under four different conditions, such as outdoors, outdoors with different clothes, outdoors with scale variation and indoors. In addition, these videos have a resolution of 120 × 160 pixels. To perform our experiments, each frame, *C*, is divided into 1,600 regions, in which each region is composed of 3 × 4 pixels. In this way, the padding is avoided. Furthermore, we select the thresholds *h* = 15, *h*′ = 6 and *α* = 6 by analyzing the speed of different human motions, as shown in Figure 5. Specifically, the proposed processing units, Π_*ec*_, computes 6 frames at each 240 ms since the video is composed of 25 frames per second. At this rate, processing units, Π_*ec*_, generate diverse firing patterns, which contain spatial and temporal information of each action. Here, we used 216 videos, which correspond to 9 people carrying out the 6 different activities in 4 different environments, 50% was used for training and the other 50% for testing and validation. Obviously, regions with significant motion (d, e and f) generate firing patterns with high number of spikes, otherwise human motions (a, b and c) produce a fewer number of spikes. Here, these patterns have helped us to create characteristic vectors.

**FIGURE 6:**
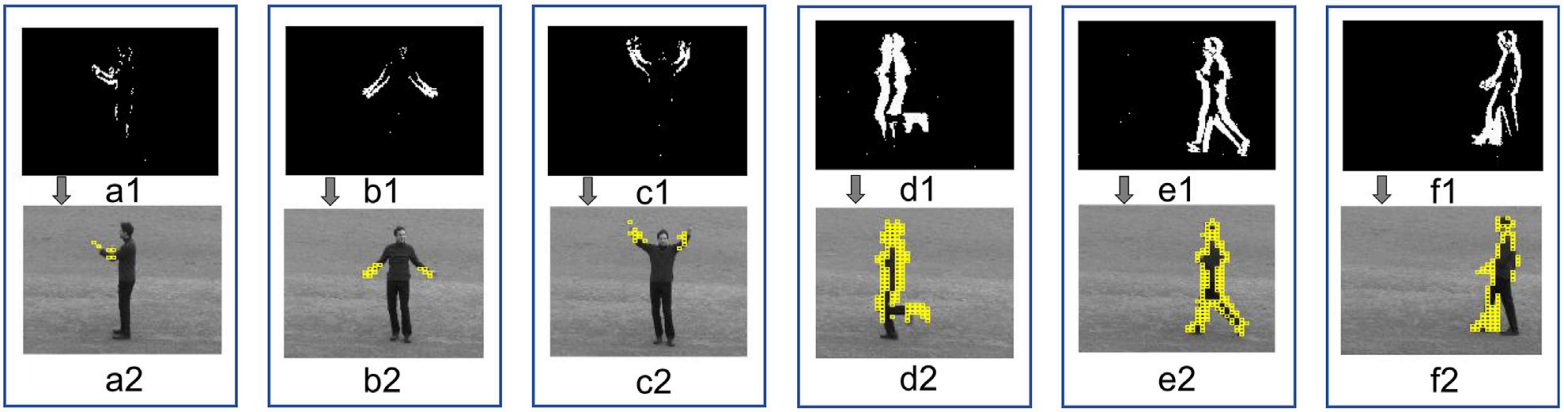
Detection of the human motion using the proposed processing units, Π_*ec*_, where **a1, b1, c1, d1, e1, f1** show the regions *R* of the motions boxing, hand clapping, hand waving, jogging, running and walking respectively, additionally **a2 b2, c2, d2, e2, f2** show which part of the human body represents the motions boxing, hand clapping, hand waving, jogging, running and walking respectively.

Our preliminary results show that the processing units, Π_*ec*_, are able to identify the human motions, as shown in Figure 6. As can be observed, images (a1, b1, c1, d1, e1 and f1) show the regions, *R*, in which the human motion is detected by the proposed processing units, Π_*ec*_, and images (a2, b2, c2, d2, e2 and f2) show which part of the human body motion moves.

Once the characteristic vectors were defined, these are used to classify different human actions. To classify these actions, we use the tool called “classification learner” of Matlab, which contains several classifiers, such as Support Vector Machine (SVM), Weighted k nearest neighbors (WKNN), Esemble Subspace Discriminant (ESD), and Esemble Bagged Trees (EBT). It should be noted that this tool contains low complexity classifiers. As a consequence, their performance in terms of the recognition accuracy is limited, as shown in Table 1. As can be observed, the use of the proposed processing units, Π_*ec*_, significantly increases the performance of these low-computational complexity classifiers. Specifically, the hierarchical organization of the proposed SN P system has shown that selective attention coordinates the neural activity to detect objects in a compact visual representation. In addition, we compare the performance of our approach and existing works, as shown in Table 2. As can be observed, the proposed human action recognition achieves the best performance reported to date. It should be noted that the existing classifiers intends to achieve high performance at the cost of increasing their computational complexity (Ghamisi et al., 2017). In our approach, we use one of the most simplest algorithm along with the proposed processing units, Π_*ec*_ to significantly improve the performance of the human action recognition system.

**TABLE 1:** Performance comparison between different classifiers of the classification learner tool.

**Classifier**	**Average Performance (%) without using** **Π**_***ec***_	**Average Performance (%) using** **Π**_***ec***_
SVM	83%	97.2%
WKNN	78%	97.2%
ESD	85%	97.7%
EBT	80%	98.1%

**TABLE 2:** Performance comparison between the proposed human action recognition system and existing algorithms.

**Author**	**Algorithm**	**Dataset**	**Real time**	**Performance (%)**
This work	SNP and SVM	KTH (Schuldt et al., 2004)	Yes	97.2%
Dash et al., (2021)	SIFT and CNN	KTH(Schuldt et al., 2004)	No	90.0%
Veeriah et al., (2015)	Differential RNN	KTH(Schuldt et al., 2004)	No	93.9%
Shi et al., (2015)	DTT. DNN	KTH (Schuldt et al., 2004)	No	95.6%
Liu et al., (2014)	MTSL	KTH (Schuldt et al., 2004)	No	96.7%
Ali and Wang, (2014)	DBN and SVM	KTH (Schuldt et al., 2004)	No	94.3%
Shu et al., (2014)	3DGab and SNN	KTH (Schuldt et al., 2004)	No	92.3%
Grushin et al., (2013)	LSTM	KTH (Schuldt et al., 2004)	No	90.7%
Ji et al., (2012)	3DCNN	KTH (Schuldt et al., 2004)	No	90.02%
Baccouche et al., (2011)	CNN and RNN	KTH (Schuldt et al., 2004)	No	94.3%

## Conclusion

In this work, we present for the first time a pre-processing method based on the spiking neural systems for efficient feature extraction in video sequences for motion detection of human actions. Specifically, we propose a basic processing unit, Π_*ec*_, with hierarchical structure inspired by the selective attention mechanism. Since the SN P systems exhibits an intrinsic parallel processing, we propose a parallel video processing system by using multiple processing units, Π_*ec*_, where each unit is implemented by mainly using comparators. As a consequence, the proposed units exhibit low computational complexity. From the engineering point of view, this potentially allows their implementation in embedded devices, where the area is limited. Part of future work is to perform more experiments by building a remote patient monitoring.

## Data Availability

The raw data supporting the conclusions of this article will be made available by the authors, without undue reservation.
